# Integrated bioinformatics analysis of differentially expressed genes in the temporomandibular joint internal derangement

**DOI:** 10.1002/cre2.768

**Published:** 2023-08-09

**Authors:** Junda Yang

**Affiliations:** ^1^ The Stomatological Hospital (College) of Xi'an Jiaotong University Shaanxi Xian China

**Keywords:** hub genes, immune cells, integrated bioinformatics analysis, temporomandibular joint internal derangement

## Abstract

**Objectives:**

This study aimed to identify significant mechanisms and potential treatments for temporomandibular joint internal derangement (TMJD) through integrated bioinformatics analysis.

**Materials and Methods:**

Gene expression data sets (GSE66864) from the Gene Expression Omnibus (GEO) database were downloaded. Differentially expressed genes (DEGs) were identified both in the treatment groups and in controls by R packages. Network analysis of protein–protein interaction (PPI) and Human Protein Atlas was used to explore DEGs' potential function. DGIdb database was utilized to gain potential drug targets.

**Results:**

In conclusion, 126 DEGs were selected for TMJD through bioinformatics analysis. Both GO and Kyoto Encyclopedia of Genes and Genomes analyses combined showed the pathways involved in TMJD. A PPI network was constructed to select the top 10 hub genes, of which five hub genes were chosen for further investigation. Moreover, the microenvironment of immune cells related to hub genes was evaluated by R packages. Macrophages play an important role in inflammation and oral‐related tumors. The Human Protein Atlas analysis indicated that the five hub genes are highly related to head and neck cancer. Finally, eight potential drugs were selected for two genes using the DGIdb database.

**Conclusion:**

Our integrated bioinformatics analysis identified DEGs in TMJD and provided potential ideas for further research and treatment approaches. However, experimental validation of the hub genes and potential drug targets is still needed. The key mechanisms of the identified genes and their potential roles as biomarkers or drug targets in TMJD require further investigation.

## 1 INTRODUCTION

Temporomandibular joint Internal derangement (TMJD) is one kind of the most complex human diseases (Granados, 1979); it is a common disease affecting more than 15% of adults, peaking in incidence among those aged 20–40 years (Gauer & Semidey, 2015). The main etiology of TMJD is complex, including biological, social, and emotional factors. TMJD is associated with primary headaches, neurological disorders, dental occlusion confusion, and a series of oral–maxillofacial diseases. Previous studies focus on the treatment of TMJD, such as Arthrocentesis versus glucocorticosteroid injection (AbdulRazzak et al., 2021). Few studies discuss the biological pathway of TMJD. As a result, currently, the molecular process associated with pathways in the TMJ is still unclear. Therefore, we aim to elucidate the biological progression of TMJD by bioinformatic analysis.

As a complex multifactorial disease, multiple pathways of hub genes and biological procession are involved in TMJD. High‐throughput sequencing technologies are increasingly used in tumor genomics and can also provide insights into nontumor diseases. Bioinformatics analysis has been shown to discover potential early diagnostic biomarkers for osteoarthritis (Cao et al., 2021). However, there is little research about TMJD on the gene expression profile and the biological pathways to explore critical signaling pathways and some essential hub genes for the process, complication, and further development of TMJD. Therefore, this study aims to figure out the hub genes and key biological pathways of TMJD, thus opening a novel perspective on this problem. Elucidating the molecular basis of TMJD can guide the development of targeted therapies and diagnostic tests for this complex disease. A better understanding of the biological mechanisms underlying TMJD may help clinicians manage the condition more effectively.

In this study, we identified differentially expressed genes (DEGs) from the Gene Expression Omnibus (GEO) database in published transcriptomic data sets of TMJD in the condylar cartilage of rabbits from the GEO database (GSE66864). DEGs that were highly concerning TMJD underwent Gene Ontology (GO), the Kyoto Encyclopedia of Genes and Genomes (KEGG) pathways, and the weighted gene coexpression network analysis (WGCNA). Protein–protein interaction (PPI) network analysis was utilized to figure out the hub genes, potential key biological procession, and signaling pathways of TMJD. Rabbit genes share similarities with human genes, including the class I major histocompatibility complex gene (Marche et al., 1985). Hence, this study utilized the Human Protein Atlas to analyze the key protein function in the human tissue, hoping to find a similar biological progression in humans. Altogether, an integrated analysis was performed in this study to identify the hub genes of TMJD and illustrate the potential biological procession and signaling pathways for the progression of TMJD. Some information can be gathered for the diagnosis, monitoring, and treatment of TMJD. Some insights may aid the diagnosis, monitoring, and treatment of TMJD, providing a novel perspective for future TMJD research.

## 2 MATERIALS AND METHODS

### 2.1 Data acquisition

The GSE66864 gene expression series matrix was downloaded from the GEO database (https://www.ncbi.nlm.nih.gov/geo/). Raw database GSE66864 was presented by the GPL13288 platform, including one control group and four treatment groups. Four rabbits were selected in one group as control (without any treatment); 16 rabbits were divided randomly into four treatment groups, which were used for histological analysis, RNA microarray analysis, and real‐time PCR at 1 week, 2 weeks, 4 weeks, and 8 weeks, respectively (Wang et al., 2015).

### 2.2 DEG identification

Background calibration, normalization, and data filter were presented to DEGs through the R software (version 4.1.3) and GEO2R (https://www.ncbi.nlm.nih.gov/geo/geo2r). DEGs with a statistically significant difference were identified through standard adjustment *p* < .05 and log fold‐change (log FC)| ≥ 2. R package “ggplot2” and “ggrepel,” were used to plot the volcano map. A heatmap was generated using TBtools (Chen et al., 2020). WGCNA was utilized to confirm the reliability of DEGs in the different samples.

### 2.3 Analysis of biological functions and signaling pathways

The GO terms and KEGG pathways of DEGs concerning the analysis of biological functions were enriched via the R package “clusterProfiler” (Yu et al., 2012). Three types of biological analyses were performed in the GO terms: biological process (BP), cellular component (CC), and molecular function (MF) in GO terms. The *p*_adj_ < .05 and *q*< .05 were set for the enriched DEGs and their further data visualization as a threshold. Gene enrichment in KEGG pathways shows similar results.

### 2.4 Construction of PPI network

In our study, we established the PPI network that contained the hub genes via the STRING database (https://cn.string-db.org/) (version 11.5) (Szklarczyk et al., 2019), and the minimum required interaction score was set at 0.4. The differentially expressed proteins were all utilized to construct PPI visualization networks and submit the DEGs by Cytoscape (Shannon et al., 2003). The top 10 DEGs were selected as nodes based on the Degree algorithm (Chin et al., 2014).

### 2.5 Analysis of immune cell infiltration

The immune cell infiltration was analyzed using cell type identification by estimating related subsets of RNA transcripts (CIBERSORT) (Newman et al., 2015). The relationship between the hub gene and the 547 genes, which distinguish 22 human immune cell phenotypes, was visualized and performed using a heatmap. The hub genes were also explored between the immune cell infiltration and human tissue. The hub genes were submitted to the Human Protein Atlas (https://www.proteinatlas.org/) to identify similarities in human tissue.

### 2.6 Drug interaction of hub genes

The DGIdb database (http://dgidb.genome.wustl.edu/) was utilized to figure out potential drug targets and potential therapeutic candidates for the upregulated hub genes. The DGldb database was established with various sources of drugs, including immunotherapy drugs and antineoplastic drugs (Cotto et al., 2018).

## 3 RESULTS

### 3.1 Identification of DEGs

One control group and four TMJD groups were contained in the GSE66864 database. We plotted a heatmap based on the expression of genes in each sample (Figure 1a). A total of 104 upregulated and 22 downregulated genes were shown in Figure 1b. Five hub genes were selected including *ITGA6, PTHLH, FPR1, ANXA8, and ATP6V0A2*, which are highly upregulated in the DEGs. The WGCNA of sample clustering showed no significant difference between each sample (Figure 1c). The soft threshold was set to 7 (*R* ^2^ = 0.8) to make the construction of the scale‐free network (Figure 1d). Several modules were identified based on average hierarchical clustering and dynamic tree clipping (Figure 1e,f). The brown‐red module had a high correlation with pathological grades.

Figure 1(a) Identification of differentially expressed genes (DEGs) in temporomandibular joint internal derangement (TMJD). Heatmap of top 500 DEGS from GSM1633547 to GSM1633566 in GSE66864. Red past represented the genes that are upregulated (up), while green parts represented the genes that are downregulated (down). (b) Volcano plot of DEGs. Red represents upregulated DEGs; blue represents downregulated DEGs; gray represents genes with no significant difference. (c) Clustering dendrogram of 20 samples. (d) Analysis of the scale‐free index with various soft‐threshold power. *R* ^2^ is equal to 0.8, which is mapped out by the red line. (e) Clustering dendrogram of all samples. (f) Network heatmap plot of the selected key genes modules and correlation between each module. NA, not available; not sig., not significant.

**Figure:**
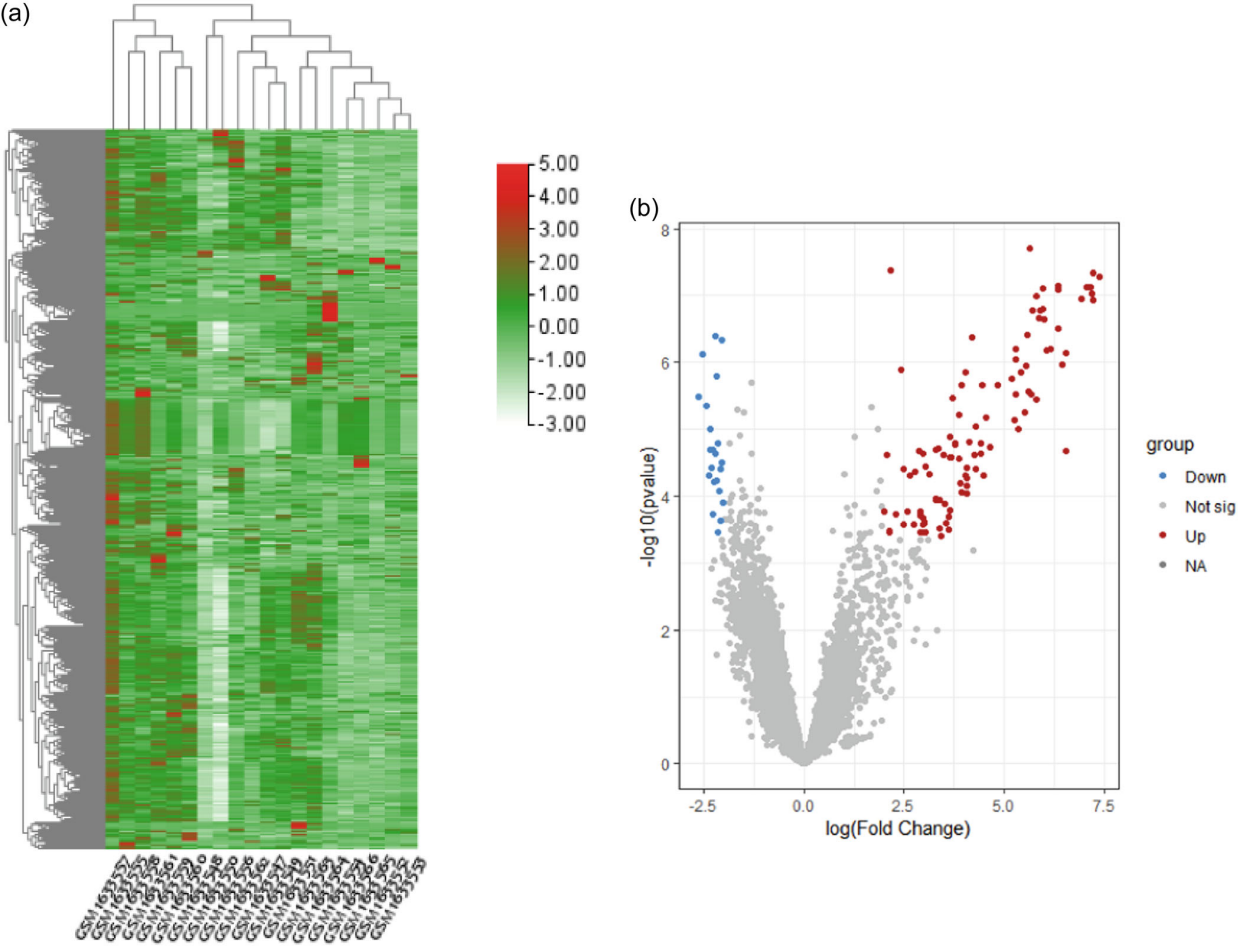

**Figure:**
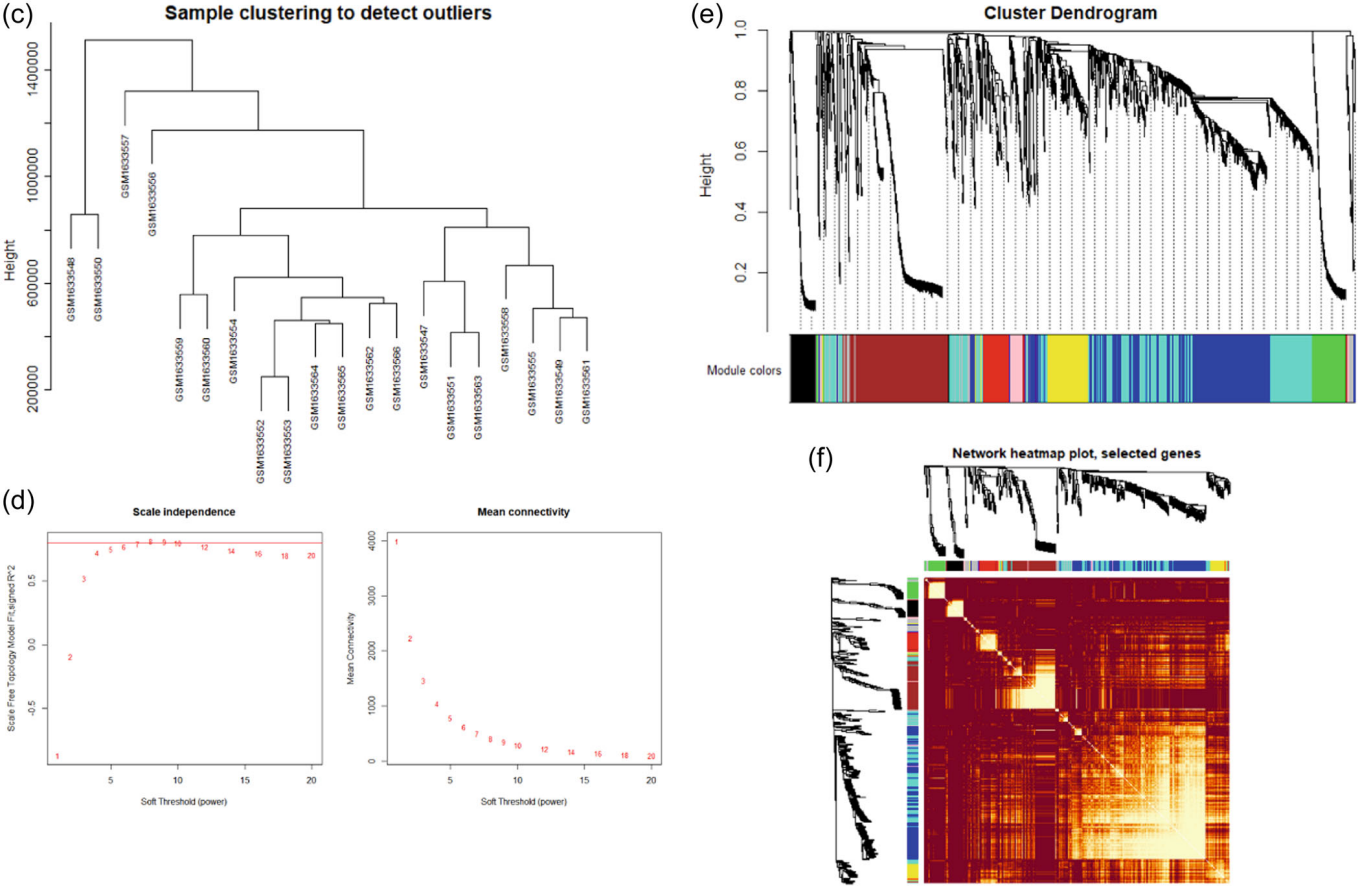

### 3.2 Analysis of biological functions and signaling pathways

**Figure 2:**
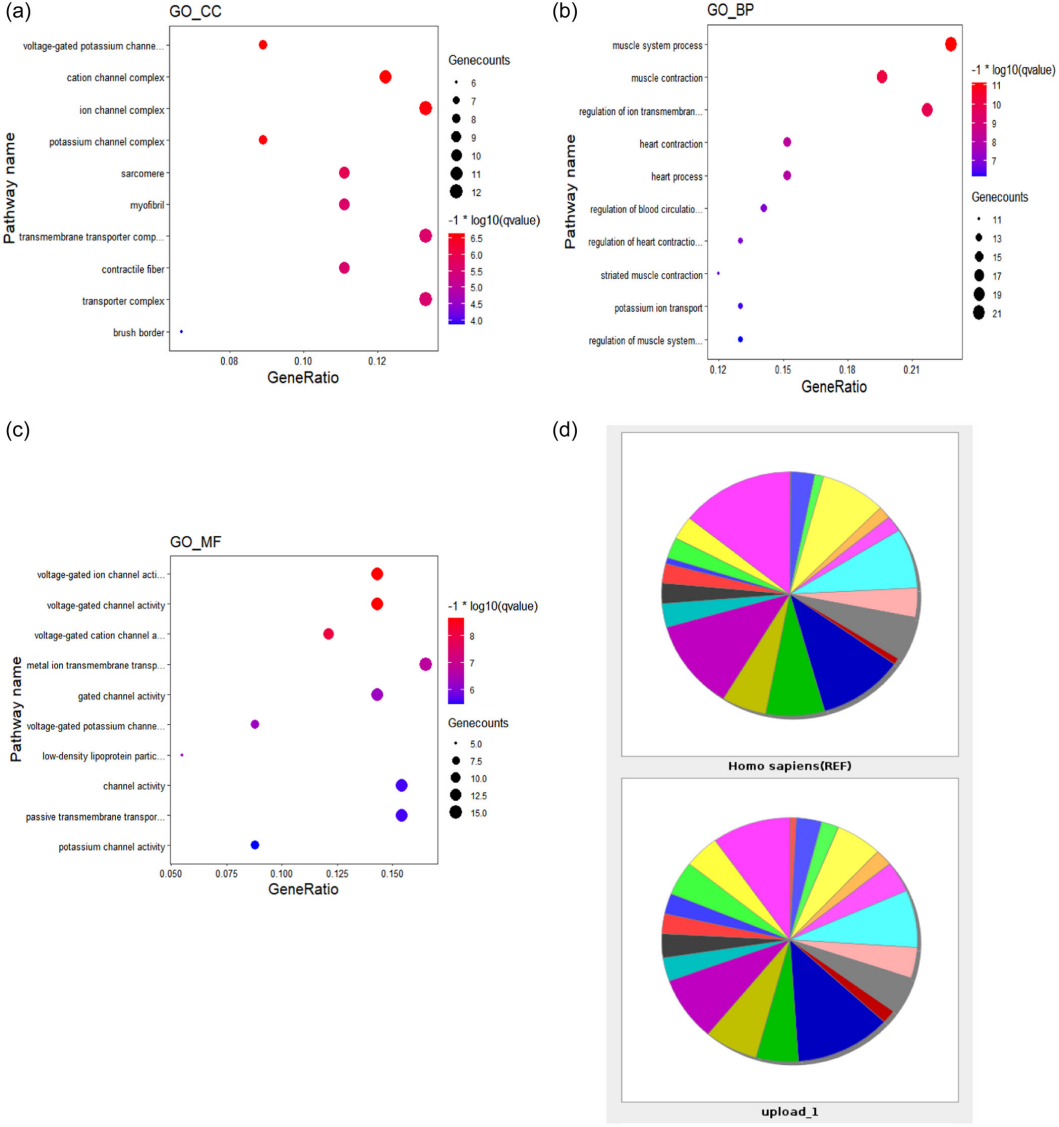
(a) Cellular component (CC), (b) biological process (BP), (c) molecular function (MF), and (d) Kyoto Encyclopedia of Genes and Genomes (KEGG) enrichment analysis. GO, Gene Ontology.

The GO enrichment analysis via dot plot showed that muscle system processes were highly associated with the BP group genes, regulation of ion transmembrane, and procession of muscle contraction (Figure 2a). In addition, the CC group genes were especially associated with the cation channel complex, ion channel complex, sarcomere, myofibril, contractile fiber, transmembrane transporter complex, and transporter complex (Figure 2b). MF groups were highly associated with voltage‐gated ion channel activity, voltage‐gated channel activity, voltage‐gated cation channel activity, metal ion transmembrane transport, gated channel activity, and passive transmembrane transport (Figure 2c). The KEGG pathway enrichment analysis shows a similar result of hub genes between human and rabbits (Figure 2d), which inspire us to associate rabbit genes obtained in this study with human genes.

### 3.3 Construction of PPI network

**Figure 3:**
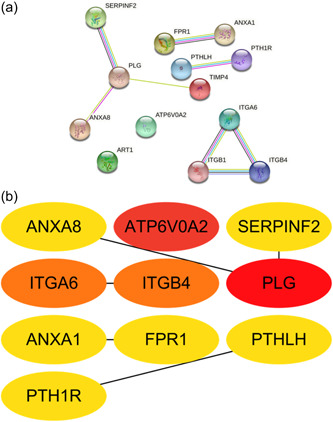
(a) Protein–protein interaction (PPI) network was constructed based on all the 126 differentially expressed genes (DEGs) by using the STRING database under the threshold of 0.4 interaction score. (b) The PPI network of DEGs in temporomandibular joint internal derangement (TMJD) (STRING). The top 10 genes included *ATP6V1A*, *ATP6V0D1*, *ATP6V1B2*, *ATP6V1D*, *ATPV1E1*, *ATP6V0A2*, *ATP6V1B1*, *ATP6V0D2*, *ATP6V0C*, and *ITGAV*, with a high degree of connections.

The 126 key genes were submitted to the String database, constructing PPI networks under the threshold of 0.4 interaction score (Figure 3a). Cytoscape software was used to illustrate the interaction result. The top 10 genes were selected by Degree, which is one of the algorithms: *ATP6V0A2, PTHLH, FPR1, ANXA8, ITGA6, PLG, SERPINF2, ATP6V0A2, ITGB4, and PTHLH1R* (Figure 3b).

### 3.4 Immune cell infiltration in TMJD

The bars depict the abundance of 22 immune cell infiltrates in the temporomandibular joint tissue samples (Figure 4a). Obviously, tumor‐associated macrophages and inflammation were detected at high levels, including T cells CD4 memory activated, B cell memory, and dendritic cells activated. The Human Protein Atlas was used to find the information about the hub genes, including *ITGA6* (Figure 4b), *FPR1* (Figure 4c), *PTHLH* (Figure 4d), *ATP6V0A2* (Figure 4e), and *ANXA8* (Figure 4f). The database shows that the *ITGA6* and *PTHLH* are highly relevant to head and neck cancer, which are specifically expressed in the tissue. Meanwhile, the *FPR1* is enriched in Immune cells (neutrophils).

Figure 4(a) Immune cell infiltration and abundance percentage of immune infiltrating in temporomandibular joint internal derangement (TMJD) tissues. (b) Percentage of *ITGA6* expression of cancer tissues from high to low. Head and neck cancer displayed the highest expression of *ITGA6*. (c) *FPR1* expression of immune cell types from high to low. Neutrophils displayed the highest expression of *FPR1*. (d) *PTHLH* expression of cancer tissues. Head and neck cancer displayed the highest expression of *PTHLH*. (e) *ATP6V0A2* expression of immune cell types from high to low. Eosinophil displayed the highest expression of *ATP6V0A2*. (f) Survival probability of *ANXA8* in patients between high‐expression group and low‐expression group. DC, dendritic cell; NK, natural killer; PBMC, peripheral blood mononuclear cell; TCGA, The Cancer Genome Atlas.

**Figure:**
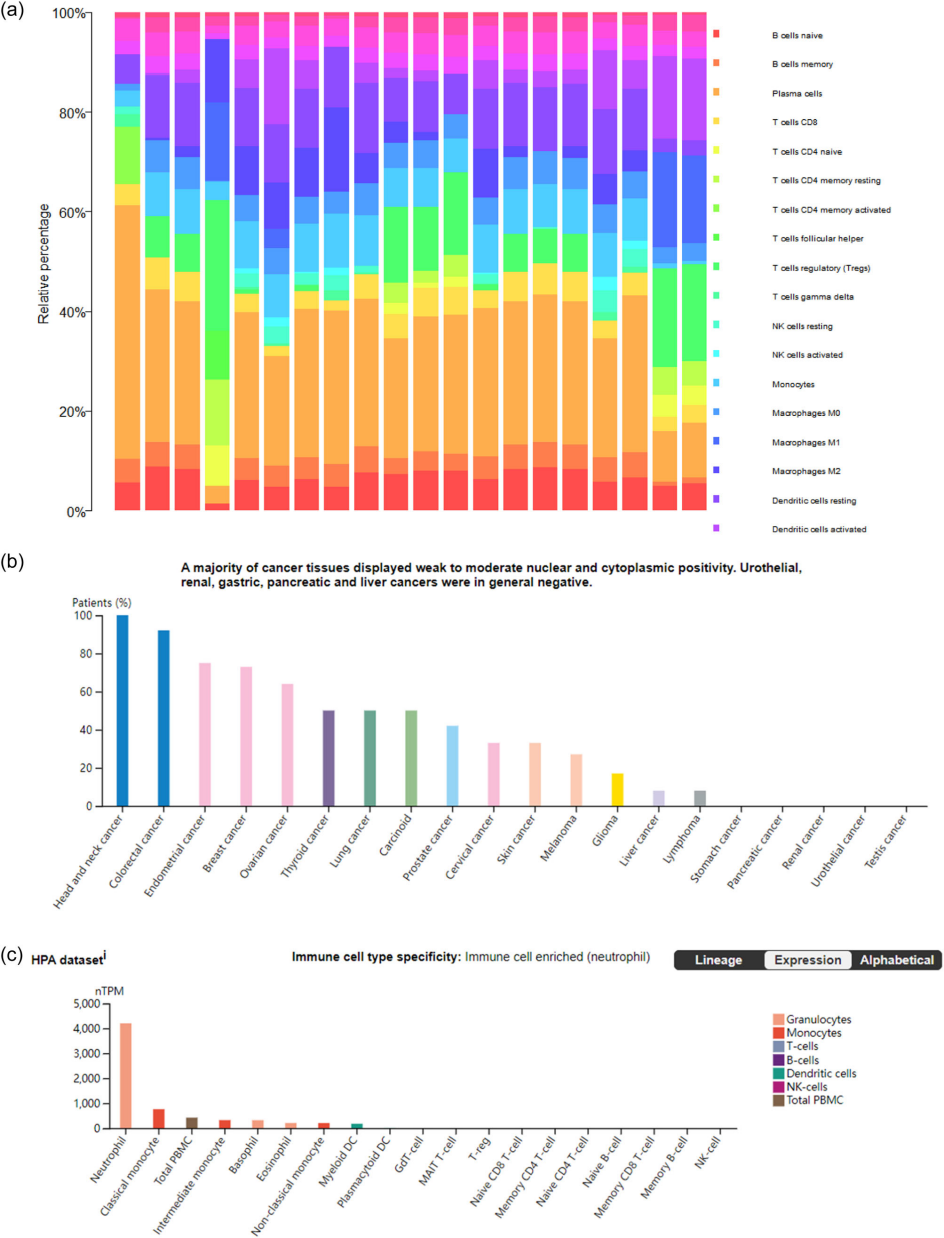

**Figure:**
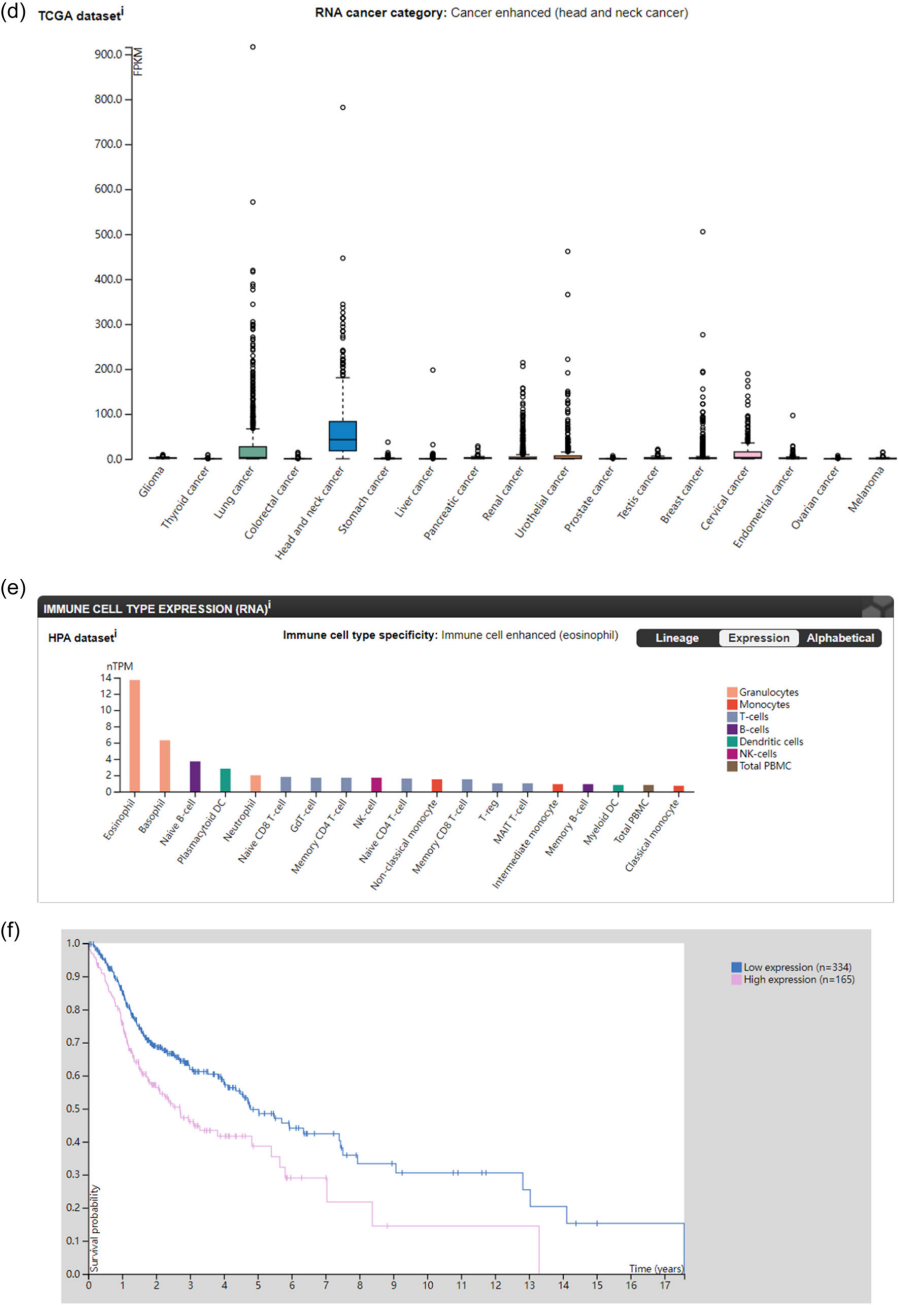

### 3.5 Drug interaction of hub genes

In our study, highly expressed hub genes were submitted to the DGIdb database (https://dgidb.org/) and eight potential drug interactions were obtained (Table 1). Two drugs interact with *PTHLH*, while five other drugs interact with *FPR1*. No drug interactions were found with *ITGA6*, *ANXA8*, and *ATP6V0A2*. The drug CAL functions as an inhibitor for *PTHLH*, and the drug CHEMBL1290251 functions as an agonist for *FPR1*.

**Table 1:** Eight potential drug interactions between DEGs.

Gene	Drug	Interaction types	Sources
PTHLH	Vinblastine		NCI
PTHLH	CAL	Inhibitor	ChemblInteractions
FPR1	Penicillin G potassium		DTC
FPR1	CHEMBL1290251	Agonist	DTC
FPR1	Phenylbutazone		DTC
FPR1	Sulfinpyrazone		DTC
FPR1	CHEMBL1290365		DTC
FPR1	Rebamipide		TTD

Abbreviation: DEG, differentially expressed gene.

## 4 DISCUSSION

TMJD is a common joint disease often associated with TMJ synovitis (Ibi, 2019). Besides, TMJD also has a noninflammatory origin. Open surgical intervention is a traditional treatment for TMJD (Buckley et al., 1993). However, the open surgery did not offer a pleasing performance in patients' experience of postoperative recovery. Given that TMJD has complex and sometimes controversial etiologies, elucidating the potential biological mechanisms of TMJD is essential to develop novel diagnostic strategies for TMJD patients.

In this study, biological analysis of a gene expression series matrix was conducted and identified 104 genes that are highly expressed and 22 genes that are downregulated between TMJDs and normal tissues. DEGs were submitted to the GO and KEGG analysis to explore the potential pathogenesis and developmental mechanisms of TMJDs. Five hub genes were selected including *ITGA6*, *PTHLH*, *FPR1*, *ANXA8*, and *ATP6V0A2*, which are upregulated in the DEGs. The upregulated genes are highly related to a muscle system process, regulation of ion transmembrane, voltage‐gated channel activity including PI3K‐Akt signaling pathway, immune responses, and inflammatory responses. Many researchers have confirmed that the PI3K‐Akt signaling pathway may be involved with TMJD caused by pressure and inflammation by increasing the cadherin‐11 in synovial fibroblasts(Wu et al., 2013). Meanwhile, some research also shows that the activation of neural pathways, for instance, the agents such as serotonin and nitric oxide mediated the pain of TMJD (Patil & Kirkwood, 2007). A PPI network was constructed by submitting the hub genes to the STRING database and Cytoscape to identify the main proteins related to TMJD.

To better illustrate the mechanism underlying TMJD pathogenesis, light was shed on the immune cell infiltration of the TMJD tissue via the R package and discovered the biological functions of these hub genes in the inflammation procession. The immune cell infiltration is intricately linked to the tumor microenvironment (Gajewski et al., 2013). Our results demonstrate that TMJD tissue had higher immune cell infiltration compared to normal tissue. The level of T‐cell CD4 memory activated was greatly enhanced. Some researchers have found that the infiltration of memory CD4+ T cells is directly implicated in rheumatoid arthritis, which is a typical arthritis with inflammation and a high amount of CD4+ T cells always releases a high level of interleukin‐17 (*IL‐17*) and tumor necrosis factor‐β (*TNF‐β*), which are the most important inflammatory factors (Chemin et al., 2019). The high frequencies of the expression of CD4+ T cells always relate to chronic inflammatory syndromes. Our study has discovered the relationship between TMJD and the infiltration of CD4+ T cells. We hypothesize that the CD4+ T cells may have a similar function in the TMJD tissues. Some research studies indicate that the TNF‐α has an impact on inhibiting the CD28 gene expression (Bryl et al., 2001). The fusion protein CTLA4 can prevent the T cells from activating by binding to CD80 and CD86 on antigen‐presenting cells and blocking cohesive the process in the engagement of CD28 (Kremer et al., 2003). According to research conducted by Joel M. Kremer et al. (2003), the upregulated hub genes may provide a new vision of biological treatment. So, the hub genes were conducted to the DGIdb database, and two and six drugs interacting with *PTHLH* and *FPR1*, respectively, were uncovered through the DGIdb database, which gives the possibility of inventing potential therapeutic candidates for TMJD.

*ITGA6* encodes a member of the integrin ⍺‐chain family of proteins, which are involved in cell surface adhesion and signaling. The α6β1 integrin may promote tumorigenesis. Some research studies show that the high level of transcriptions of the *ITGA6* can be specifically found in the tumor tissues, displaying an oral‐cancer‐related biomarker (Lo et al., 2012). On the contrary, the low expression of *ITGA6* can impair the activity of the PI3K/AKT pathway (Chen & Zhang, 2022) to exert a tumor‐suppressive function. *ITGA6* can also promote cell invasion by changing the microenvironment of the extracellular matrix (ECM) (You et al., 2021), suggesting a new way for oral cancer treatment by silencing the molecule. Meanwhile, *ITGA6* is involved in macrophage function, particularly migration and M2 activation (Sima et al., 2016), and the mechanisms of proinflammatory cytokines, which are derived from M1 and M2 macrophages. Macrophages' activation can be inhibited by IL‐37 through the NLRP3 pathway and by suppressing the lipopolysaccharide and interferon‐γ‐induced extracellular signal‐regulated kinase (ERK) and nuclear factor‐κB (NF‐κB) activation in human M1 macrophages (Luo et al., 2020).

The *PTHLH* gene is related to the development of endochondral bone. Studies showed that *PTHLH* is highly expressed in oral cancer (Lv et al., 2014) and tongue cancer (Suwa et al., 2014). PTHrP, which is the protein encoded by *PTHLH*, is mostly produced and expressed by many tumor tissues and just a few normal cells. The normal tissues have a low concentration of PTHrP (Park & McCauley, 2012). Cell proliferation was reported to be highly associated with the upregulation of the *PTHLH* gene (Hameetman et al., 2005). Estrogen can also affect *PTHLH* expression in the tissue which may enhance the tumor growth in the cartilaginous tissue. Some report also shows that Emodin can suppress the *PTHLH* expression in related cancer by suppressing the TCF4/TWIST1‐induced complex (Fang et al., 2022). In this study, the *PTHLH* is upregulated in the DEGs; we can infer that the high expression of this gene is linked to oral‐related cancer.

The *FPR1* gene found in mammalian phagocytic cells encodes a G‐protein‐coupled receptor. The protein plays an important role in host defense and inflammation. FPRs are expressed in nearly all kinds of immune cells, including neutrophils, macrophages, and natural killers. Among those, the neutrophils were mostly influenced by the *FPR1* gene, attracting more leukocytes to migrate to the sites of inflammation (Leslie et al., 2020). Annexin A1 (*ANXA1*) is a ligand of *FPR1* relevant for cancer immunosurveillance (Vacchelli et al., 2020). In this study, *FPR1* is highly expressed in the temporal–mandibular joint tissue, which may cause inflammation by leukocyte migration. The high expression of *FPR1* has also been proven to activate biological procession that contributed to chronic inflammation such as ERK, mitogen‐activated protein kinase, NF‐κB, and signal transducer and activator of transcription 3 (Cao & Zhang, 2018). The *FPR1* expression can predict early cancer and function as a pharmacologic target for innate immune responses (Liu et al., 2012); it plays a significant role in phagocyte accumulation and promotes innate immunity. High *ANXA8* expression suggests that it may act as a receptor for *FPR1*. Further research is needed to test this hypothesis.

*ANXA8*, the gene encodes a member of the annexin protein family that binds to cell membranes. Rosenbaum et al. (2011) discover that the *ANXA8* lacks the ability to bind to phosphatidylserine (PS) and apoptotic cells. If more apoptotic cells are cleared and identified, it may suppress the inflammation reaction by preventing the release of detrimental contents in cells. The loss of PS in the cells is the biomarker that the early apoptotic cells contained. The receptors in the phagocyte cell surface consist of the family members of scavenger receptors; moreover, TIM1 and TIM4 can also bind to PS straight on dying cells. *ANXA5* especially interacted with PS, which is a specific partner (van Genderen et al., 2006), while *ANXA8* lacks the ability, which may explain why the high expression of *ANAX8* is the factor of inflammation and cell apoptosis. The high expression of *ANXA8* is also associated with oral squamous cell carcinoma (OSCC) by metastasizing the cervical lymph node, suggesting potential treatment avenues. Some reports detected the expression of *ANXA8* expression in the different groups of cervical lymph nodes (Oka et al., 2016) and found that the *ANAX8* messenger RNA expression was rapidly detected in the metastasis‐positive group and rarely detected in the normal group. In some reports, *ANXA8* was frequently overexpressed in pancreatic cancer (Karanjawala et al., 2008), but its role in other cancers, including OSCC, is unclear, including OSCC and other oral‐related cancer. In our study, we also found the survival probability between high‐ and low‐expression patients who have oral cancers. The high‐expression group has a lower survival probability. All of these pieces of evidences may indicate that the *ANXA8* may play a crucial role in inflammation, cell apoptosis, and oral cancer. Further research is needed to validate these mechanisms.

*ATP6V0A2*, the gene encodes an enzyme that contributed to the formation of acid microenvironment in cells. Studies suggest *ATP6V0A2* upregulation triggers macrophage transport, initiating an inflammatory state in cells (Jaiswal et al., 2012). During the window of implantation, the expression of V‐ATPase has an important function in the communication between cells and trophoblast invasion (Satokata et al., 1995). Moreover, *ATP6V0A2* may also relate to cancer cell ECM and control tumor metastasis (Katara et al., 2018) by damaging the ECM proteins by way of defective glycosylation and producing the compromised ECM. PHY34, a synthetic small molecule can target and inhibit the *ATP6V0A2* subunit of V‐ATPase (Salvi et al., 2022) and significantly reduce tumor burden. ATP6V0A2 also expresses in numerous immune cells, especially highly expresses in eosinophils according to our research through the Human Protein Atlas.

Several limitations still exist in this study. First, we obtained the data from the GEO database in this study, while there is a lack of clinical experimental research certifications for hub genes. A small number of samples with a total of one control group and four treatment groups are presented in our study, for the relative biological process of the disease still remained undiscovered. Second, our integrative bioinformatics approach requires further experimental validation to fully explore TMJD pathogenesis. However, our study provides insights into potential molecular mechanisms, biomarkers, and drugs for TMJD. We plan experimental confirmation of gene expression changes and further clinical investigation of DEGs to develop new treatments and strategies for TMJD. Despite these caveats, our study gives out further exploration into the significant molecular mechanisms of TMJD, as well as searches for potential biomarkers and therapeutic candidate drugs, providing novel treatment and biological targets for TMJD patients. We planned to perform more experiments to confirm the process of gene expression. Further clinical investigations on the DEGs may help develop the treatment and discover new diagnostic and therapeutic strategies for TMJD.

## 5 CONCLUSION

This study provides new insights into the biological factors underlying TMJD. Using integrative bioinformatics analysis, we identified five hub genes (*ITGA6*, *PTHLH*, *FPR1*, *ANXA8*, and *ATP6V0A2*) that likely contribute to TMJD pathogenesis. These genes were found to be involved in processes including inflammation, ECM regulation, immune responses, and tumorigenesis, shedding light on potential mechanisms of TMJD.

We also identified potential drugs that target two of the hub genes, representing candidate therapeutics for TMJD. However, further experimental validation is needed to confirm the roles of these genes in TMJD and evaluate the efficacy of potential drug treatments.

In summary, this study represents an important first step in understanding the biological processes and molecular mechanisms involved in TMJD. Future research should experimentally test the findings and advance the development of effective TMJD treatments and diagnostic strategies. With additional investigation and confirmation, our work may help to improve TMJD management and patient outcomes.

## AUTHOR CONTRIBUTIONS

**Junda Yang**: Conceptualization; methodology; software; data curation; writing—original draft preparation.

## CONFLICT OF INTEREST STATEMENT

The author declares no conflict of interest.

## Data Availability

Data are openly available in a public repository that issues data sets with DOIs.

## References

[cre2768-bib-0001] AbdulRazzak, N. J. , Sadiq, J. A. , & Jiboon, A. T. (2021, June). Arthrocentesis versus glucocorticosteroid injection for internal derangement of temporomandibular joint. Oral and Maxillofacial Surgery, 25(2), 191–197. 32870434 10.1007/s10006-020-00901-3

[cre2768-bib-0002] Bryl, E. , Vallejo, A. N. , Weyand, C. M. , & Goronzy, J. (2001, September 15). Down‐regulation of CD28 expression by TNF‐α. The Journal of Immunology, 167(6), 3231–3238. 10.4049/jimmunol.167.6.3231 11544310

[cre2768-bib-0003] Buckley, M. J. , Merrill, R. G. , & Braun, T. W. (1993). Surgical management of internal derangement of the temporomandibular joint. Journal of Oral and Maxillofacial Surgery, 51, 20–27. 8419583 10.1016/0278-2391(93)90006-y

[cre2768-bib-0004] Cao, G. , & Zhang, Z. (2018, November 16). FPR1 mediates the tumorigenicity of human cervical cancer cells. Cancer Management and Research, 10, 5855–5865. 10.2147/CMAR.S182795 30510453 PMC6248370

[cre2768-bib-0005] Cao, J. , Ding, H. , Shang, J. , Ma, L. , Wang, Q. , & Feng, S. (2021). Weighted gene co‐expression network analysis reveals specific modules and hub genes related to immune infiltration of osteoarthritis. Annals of Translational Medicine, 9, 1525. 34790731 10.21037/atm-21-4566 PMC8576690

[cre2768-bib-0006] Chemin, K. , Gerstner, C. , & Malmström, V. (2019, March 12). Effector functions of CD4+ T cells at the site of local autoimmune inflammation—Lessons from rheumatoid arthritis. Frontiers in immunology, 10, 353. 10.3389/fimmu.2019.00353 30915067 PMC6422991

[cre2768-bib-0007] Chen, C. , Chen, H. , Zhang, Y. , Thomas, H. R. , Frank, M. H. , He, Y. , & Xia, R. (2020). TBtools: An integrative toolkit developed for interactive analyses of Big Biological Data. Molecular Plant, 13(8), 1194–1202. 32585190 10.1016/j.molp.2020.06.009

[cre2768-bib-0008] Chen, M. , & Zhang, J. (2022, April). miR‐186‐5p inhibits the progression of oral squamous cell carcinoma by targeting ITGA6 to impair the activity of the PI3K/AKT pathway. Journal of Oral Pathology and Medicine, 51(4), 322–331. 10.1111/jop.13288 35201653

[cre2768-bib-0009] Chin, C. H. , Chen, S. H. , Wu, H. H. , Ho, C. W. , Ko, M. T. , & Lin, C. Y. (2014). cytoHubba: Identifying hub objects and sub‐networks from complex interactome. BMC Systems Biology, 8(Suppl. 4), S11. 25521941 10.1186/1752-0509-8-S4-S11 PMC4290687

[cre2768-bib-0010] Cotto, K. C. , Wagner, A. H. , Feng, Y. Y. , Kiwala, S. , Coffman, A. C. , Spies, G. , Wollam, A. , Spies, N. C. , Griffith, O. L. , & Griffith, M. (2018). DGIdb 3.0: A redesign and expansion of the drug‐gene interaction database. Nucleic Acids Research, 46, D1068–D1073. 29156001 10.1093/nar/gkx1143 PMC5888642

[cre2768-bib-0011] Fang, X. Q. , Kim, Y. S. , Lee, Y. M. , Lee, M. , Lim, W. J. , Yim, W. J. , Han, M. W. , & Lim, J. H. (2022, April 5). *Polygonum cuspidatum* extract (Pc‐Ex) containing Emodin suppresses lung cancer‐induced cachexia by suppressing TCF4/TWIST1 complex‐induced PTHrP expression. Nutrients, 14(7):1508. 10.3390/nu14071508 35406121 PMC9002362

[cre2768-bib-0012] Gajewski, T. F. , Schreiber, H. , & Fu, Y. X. (2013, October). Innate and adaptive immune cells in the tumor microenvironment. Nature Immunology, 14(10), 1014–1022. 10.1038/ni.2703 24048123 PMC4118725

[cre2768-bib-0013] Gauer, R. L. , & Semidey, M. J. (2015, March 15). Diagnosis and treatment of temporomandibular disorders. American Family Physician, 91(6), 378–386. 25822556

[cre2768-bib-0014] van Genderen, H. , Kenis, H. , Lux, P. , Ungeth, L. , Maassen, C. , Deckers, N. , Narula, J. , Hofstra, L. , & Reutelingsperger, C. (2006). In vitro measurement of cell death with the annexin A5 affinity assay. Nature Protocols, 1(1), 363–367. 10.1038/nprot.2006.55 17406257

[cre2768-bib-0015] Granados, J. I. (1979). The influence of the loss of teeth and attrition on the articular eminence. The Journal of Prosthetic Dentistry, 42, 78–85. 379311 10.1016/0022-3913(79)90333-0

[cre2768-bib-0016] Hameetman, L. , Kok, P. , Eilers, P. H. C. , Cleton‐Jansen, A. M. , Hogendoorn, P. C. W. , & Bovée, J. V. M. G. (2005, April). The use of Bcl‐2 and PTHLH immunohistochemistry in the diagnosis of peripheral chondrosarcoma in a clinicopathological setting. Virchows Archives, 446(4), 430–437. 10.1007/s00428-005-1208-4 15744499

[cre2768-bib-0017] Ibi, M. (2019). Inflammation and temporomandibular joint derangement. Biological and Pharmaceutical Bulletin, 42(4), 538–542. 10.1248/bpb.b18-00442 30930413

[cre2768-bib-0018] Jaiswal, M. K. , Mallers, T. M. , Larsen, B. , Kwak‐Kim, J. , Chaouat, G. , Gilman‐Sachs, A. , & Beaman, K. D. (2012, May). V‐ATPase upregulation during early pregnancy: A possible link to establishment of an inflammatory response during preimplantation period of pregnancy. Reproduction, 143(5), 713–725. 10.1530/REP-12-0036 22454532

[cre2768-bib-0019] Karanjawala, Z. E. , Illei, P. B. , Ashfaq, R. , Infante, J. R. , Murphy, K. , Pandey, A. , Schulick, R. , Winter, J. , Sharma, R. , Maitra, A. , Goggins, M. , & Hruban, R. H. (2008, February). New markers of pancreatic cancer identified through differential gene expression analyses: Claudin 18 and annexin A8. American Journal of Surgical Pathology, 32(2), 188–196. 10.1097/PAS.0b013e31815701f3 18223320 PMC2678811

[cre2768-bib-0020] Katara, G. K. , Kulshrestha, A. , Mao, L. , Wang, X. , Sahoo, M. , Ibrahim, S. , Pamarthy, S. , Suzue, K. , Shekhawat, G. S. , Gilman‐Sachs, A. , & Beaman, K. D. (2018, February). Mammary epithelium‐specific inactivation of V‐ATPase reduces stiffness of extracellular matrix and enhances metastasis of breast cancer. Molecular Oncology, 12(2), 208–223. 10.1002/1878-0261.12159 29178186 PMC5792725

[cre2768-bib-0021] Kremer, J. M. , Westhovens, R. , Leon, M. , Di Giorgio, E. , Alten, R. , Steinfeld, S. , Russell, A. , Dougados, M. , Emery, P. , Nuamah, I. F. , Williams, G. R. , Becker, J. C. , Hagerty, D. T. , & Moreland, L. W. (2003, November 13). Treatment of rheumatoid arthritis by selective inhibition of T‐cell activation with fusion protein CTLA4Ig. New England Journal of Medicine, 349(20), 1907–1915. 10.1056/NEJMoa035075 14614165

[cre2768-bib-0022] Leslie, J. , Millar, B. J. M. , Del Carpio Pons, A. , Burgoyne, R. A. , Frost, J. D. , Barksby, B. S. , Luli, S. , Scott, J. , Simpson, A. J. , Gauldie, J. , Murray, L. A. , Finch, D. K. , Carruthers, A. M. , Ferguson, J. , Sleeman, M. A. , Rider, D. , Howarth, R. , Fox, C. , Oakley, F. , … Borthwick, L. A. (2020). FPR‐1 is an important regulator of neutrophil recruitment and a tissue‐specific driver of pulmonary fibrosis. JCI Insight, 5(4), e125937. 10.1172/jci.insight.125937 32102985 PMC7101152

[cre2768-bib-0023] Liu, M. , Zhao, J. , Chen, K. , Bian, X. , Wang, C. , Shi, Y. , & Wang, J. M. (2012, November). G protein‐coupled receptor FPR1 as a pharmacologic target in inflammation and human glioblastoma. International Immunopharmacology, 14(3), 283–288. 10.1016/j.intimp.2012.07.015 22863814 PMC3547636

[cre2768-bib-0024] Lo, W. Y. , Wang, H. J. , Chiu, C. W. , & Chen, S. F. (2012, December 21). miR‐27b‐regulated TCTP as a novel plasma biomarker for oral cancer: From quantitative proteomics to post‐transcriptional study. Journal of Proteomics, 77, 154–166. 10.1016/j.jprot.2012.07.039 22902387

[cre2768-bib-0025] Luo, P. , Peng, S. , Yan, Y. , Ji, P. , & Xu, J. (2020, October 1). IL‐37 inhibits M1‐like macrophage activation to ameliorate temporomandibular joint inflammation through the NLRP3 pathway. Rheumatology, 59(10), 3070–3080. 10.1093/rheumatology/keaa192 32417913

[cre2768-bib-0026] Lv, Z. , Wu, X. , Cao, W. , Shen, Z. , Wang, L. , Xie, F. , Zhang, J. , Ji, T. , Yan, M. , & Chen, W. (2014, December 18). Parathyroid hormone‐related protein serves as a prognostic indicator in oral squamous cell carcinoma. Journal of Experimental & Clinical Cancer Research, 33(1), 100. 10.1186/s13046-014-0100-y 25539663 PMC4393566

[cre2768-bib-0027] Marche, P. N. , Tykocinski, M. L. , Max, E. E. , & Kindt, T. J. (1985). Structure of a functional rabbit class I MHC gene: Similarity to human class I genes. Immunogenetics, 21(1), 71–82. 3917974 10.1007/BF00372243

[cre2768-bib-0028] Newman, A. M. , Liu, C. L. , Green, M. R. , Gentles, A. J. , Feng, W. , Xu, Y. , Hoang, C. D. , Diehn, M. , & Alizadeh, A. A. (2015). Robust enumeration of cell subsets from tissue expression profiles. Nature Methods, 12, 453–457. 25822800 10.1038/nmeth.3337 PMC4739640

[cre2768-bib-0029] Oka, R. , Nakashiro, K. , Goda, H. , Iwamoto, K. , Tokuzen, N. , & Hamakawa, H. (2016, January 26). Annexin A8 is a novel molecular marker for detecting lymph node metastasis in oral squamous cell carcinoma. Oncotarget, 7(4), 4882–4889. 10.18632/oncotarget.6639 26700817 PMC4826250

[cre2768-bib-0030] Park, S. I. , & McCauley, L. K. (2012). Nuclear localization of parathyroid hormone‐related peptide confers resistance to anoikis in prostate cancer cells. Endocrine‐Related Cancer, 19, 243–254. 10.1530/ERC-11-0278 22291434 PMC3593272

[cre2768-bib-0031] Patil, C. S. , & Kirkwood, K. L. (2007, September). p38 MAPK signaling in oral‐related diseases. Journal of Dental Research, 86(9), 812–825. 10.1177/154405910708600903 17720848

[cre2768-bib-0032] Rosenbaum, S. , Kreft, S. , Etich, J. , Frie, C. , Stermann, J. , Grskovic, I. , Frey, B. , Mielenz, D. , Pöschl, E. , Gaipl, U. , Paulsson, M. , & Brachvogel, B. (2011, February 18). Identification of novel binding partners (annexins) for the cell death signal phosphatidylserine and definition of their recognition motif. Journal of Biological Chemistry, 286(7), 5708–5716. 10.1074/jbc.M110.193086 21131363 PMC3037683

[cre2768-bib-0033] Salvi, A. , Young, A. N. , Huntsman, A. C. , Pergande, M. R. , Korkmaz, M. A. , Rathnayake, R. A. , Mize, B. K. , Kinghorn, A. D. , Zhang, X. , Ratia, K. , Schirle, M. , Thomas, J. R. , Brittain, S. M. , Shelton, C. , Aldrich, L. N. , Cologna, S. M. , Fuchs, J. R. , & Burdette, J. E. (2022, January 10). PHY34 inhibits autophagy through V‐ATPase V0A2 subunit inhibition and CAS/CSE1L nuclear cargo trafficking in high grade serous ovarian cancer. Cell Death and Disease, 13(1), 45. 10.1038/s41419-021-04495-w 35013112 PMC8748433

[cre2768-bib-0034] Satokata, I. , Benson, G. , & Maas, R. (1995). Sexually dimorphic sterility phenotypes in HoxalO‐deficient mice. Nature, 374, 460–463. 10.1038/374460a0 7700356

[cre2768-bib-0035] Shannon, P. , Markiel, A. , Ozier, O. , Baliga, N. S. , Wang, J. T. , Ramage, D. , Amin, N. , Schwikowski, B. , & Ideker, T. (2003). Cytoscape: A software environment for integrated models of biomolecular interaction networks. Genome Research, 13, 2498–2504. 14597658 10.1101/gr.1239303 PMC403769

[cre2768-bib-0036] Sima, C. , Cheng, Q. , Rautava, J. , Levesque, C. , Sherman, P. , & Glogauer, M. (2016, April). Identification of quantitative trait loci influencing inflammation‐mediated alveolar bone loss: Insights into polygenic inheritance of host–biofilm disequilibria in periodontitis. Journal of Periodontal Research, 51(2), 237–249. 10.1111/jre.12303 26126603

[cre2768-bib-0037] Suwa, H. , Hirano, M. , Kawarada, K. , Nagayama, M. , Ehara, M. , Muraki, T. , Shisa, H. , Sugiyama, A. , Sugimoto, M. , Hiai, H. , Kitano, M. , & Tanuma, J. I. (2014, January). Pthlh, a promising cancer modifier gene in rat tongue carcinogenesis. Oncology Reports, 31(1), 3–12. 10.3892/or.2013.2859 24253735 PMC3868494

[cre2768-bib-0038] Szklarczyk, D. , Gable, A. L. , Lyon, D. , Junge, A. , Wyder, S. , HuertaCepas, J. , Simonovic, M. , Doncheva, N. T. , Morris, J. H. , Bork, P. , Jensen, L. J. , & Mering, C. (2019). STRING v11: Protein–protein association networks with increased coverage, supporting functional discovery in genome‐wide experimental datasets. Nucleic Acids Research, 47(D1), D607–D613. 30476243 10.1093/nar/gky1131 PMC6323986

[cre2768-bib-0039] Vacchelli, E. , Le Naour, J. , & Kroemer, G. (2020, April 30). The ambiguous role of FPR1 in immunity and inflammation. Oncoimmunology, 9(1), 1760061. 10.1080/2162402X.2020.1760061 32391192 PMC7199809

[cre2768-bib-0040] Wang, S. , Xu, G. , Deng, L. , & Gu, Z. (2015, July 11). Transcriptomic study on the impact of temporomandibular joint internal derangement in the condylar cartilage of rabbits. Genomics Data, 5, 364–365. 26484287 10.1016/j.gdata.2015.06.034 PMC4583689

[cre2768-bib-0041] Wu, M. , Xu, T. , Zhou, Y. , Lu, H. , & Gu, Z. (2013, October). Pressure and inflammatory stimulation induced increase of cadherin‐11 is mediated by PI3K/Akt pathway in synovial fibroblasts from temporomandibular joint. Osteoarthritis and Cartilage, 21(10), 1605–1612. 10.1016/j.joca.2013.07.015 23916685

[cre2768-bib-0042] You, G. R. , Chang, J. T. , Li, Y. L. , Chen, Y. J. , Huang, Y. C. , Fan, K. H. , Chen, Y. C. , Kang, C. J. , & Cheng, A. J. (2021, July 9). Molecular interplays between cell invasion and radioresistance that lead to poor prognosis in head–neck cancer. Frontiers in Oncology, 11, 681717. 10.3389/fonc.2021.681717 34307149 PMC8299304

[cre2768-bib-0043] Yu, G. , Wang, L. G. , Han, Y. , & He, Q. Y. (2012). clusterProfiler: An R package for comparing biological themes among gene clusters. OMICS: A Journal of Integrative Biology, 16, 284–287. 22455463 10.1089/omi.2011.0118 PMC3339379

